# Supplementary figures and images for: MLL3 regulates the CDKN2A tumor suppressor locus in liver cancer
Source: eLife. 2023 Jun 1;12:e80854. doi: 10.7554/eLife.80854 (PMC10279454; doi:10.7554/eLife.80854)

Figure 1—figure supplement 2B Source files

*Kmt2c* locus  
amplicon

*Trp53* locus  
amplicon

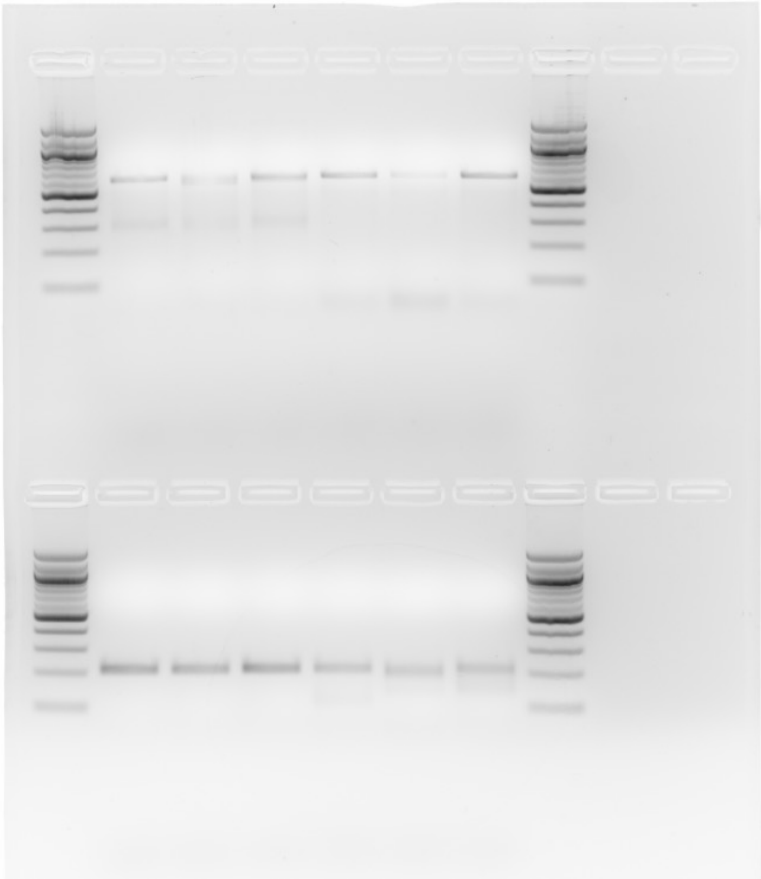

Supplement: Figure 1—figure supplement 2—source data 1. [file elife-80854-fig1-figsupp2-data1.pdf]
