# Supplementary material for: MLL3 regulates the CDKN2A tumor suppressor locus in liver cancer
Source: eLife. 2023 Jun 1;12:e80854. doi: 10.7554/eLife.80854 (PMC10279454; doi:10.7554/eLife.80854)

Figure 4—figure supplement 1A Source files

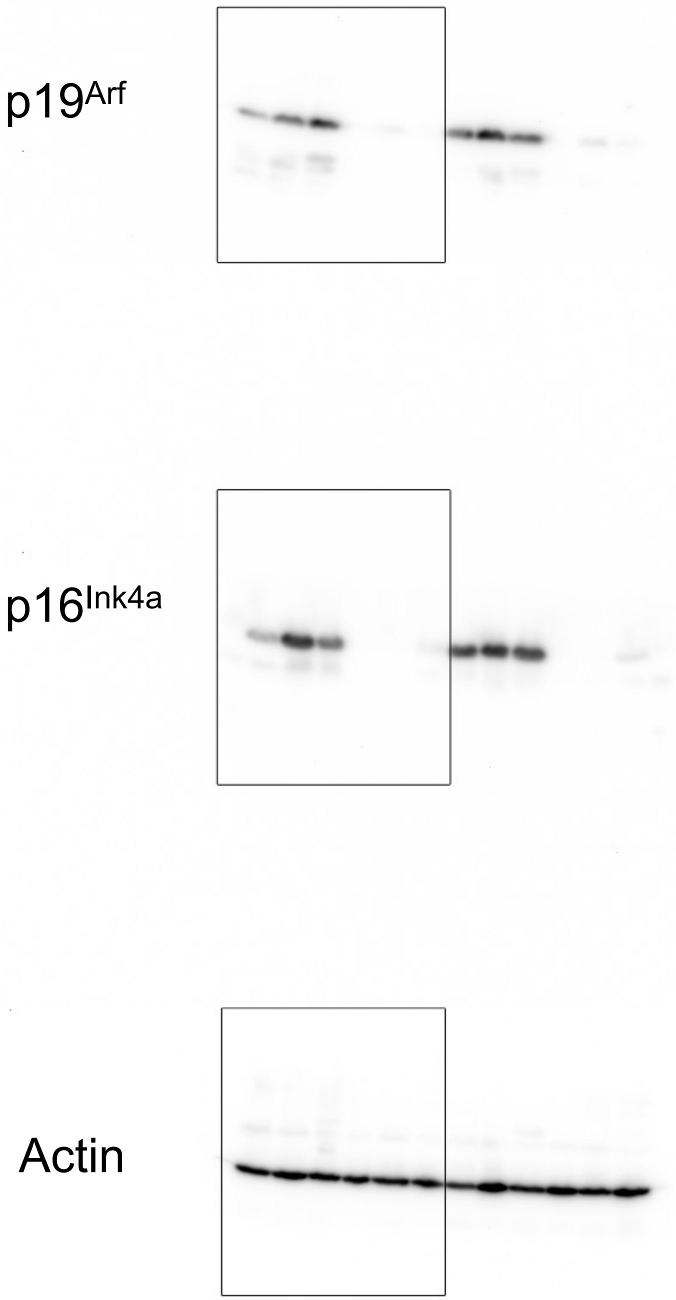

Figure 4—figure supplement 1C Source files

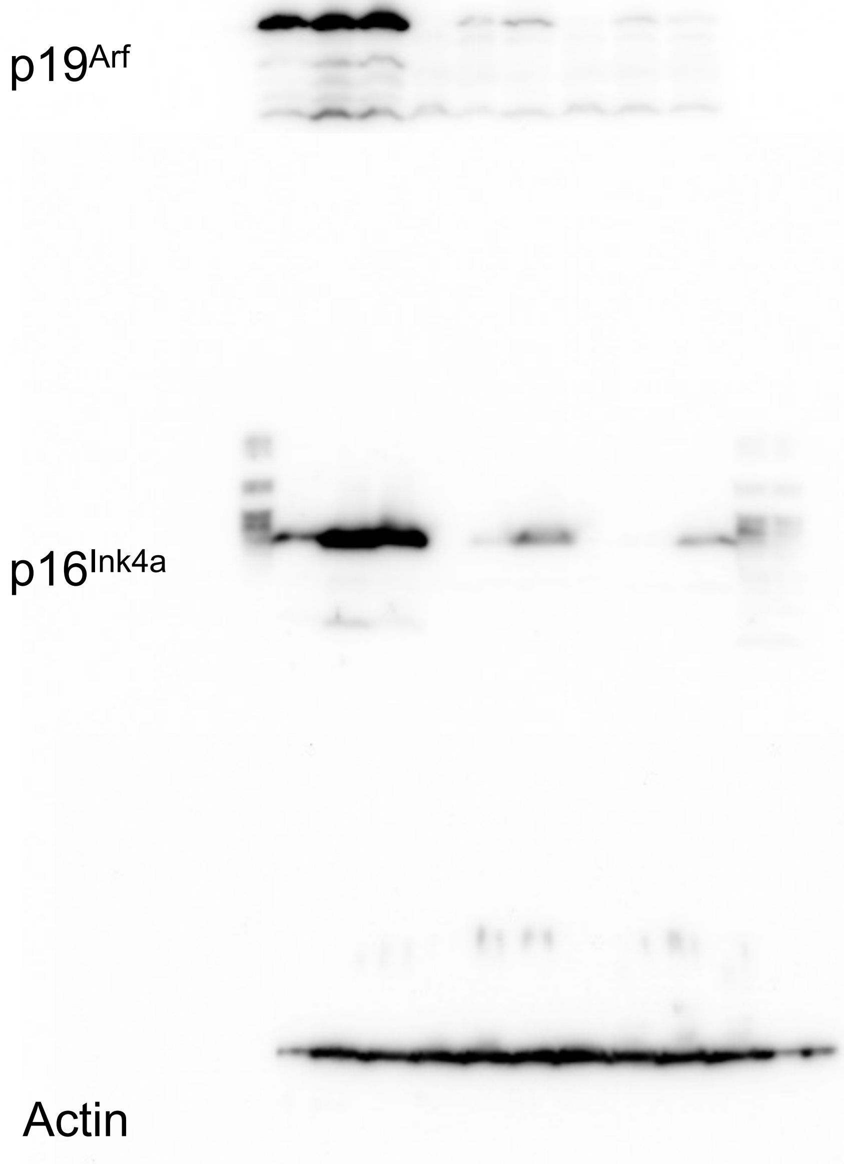

Figure 4—figure supplement 1D Source files

p19^{Arf}

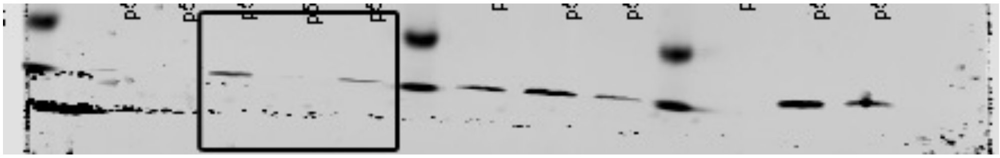

Actin

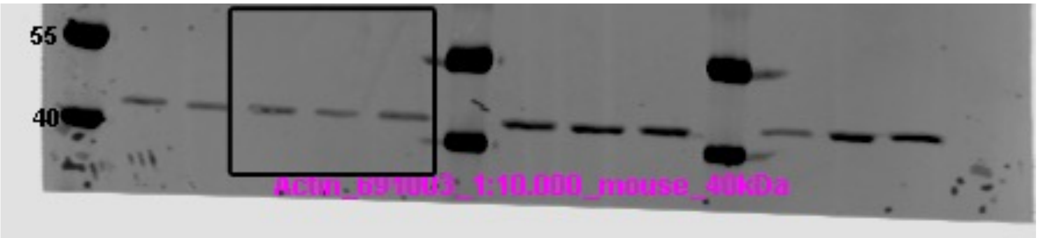

Figure 4—figure supplement 1E Source files

Axin1

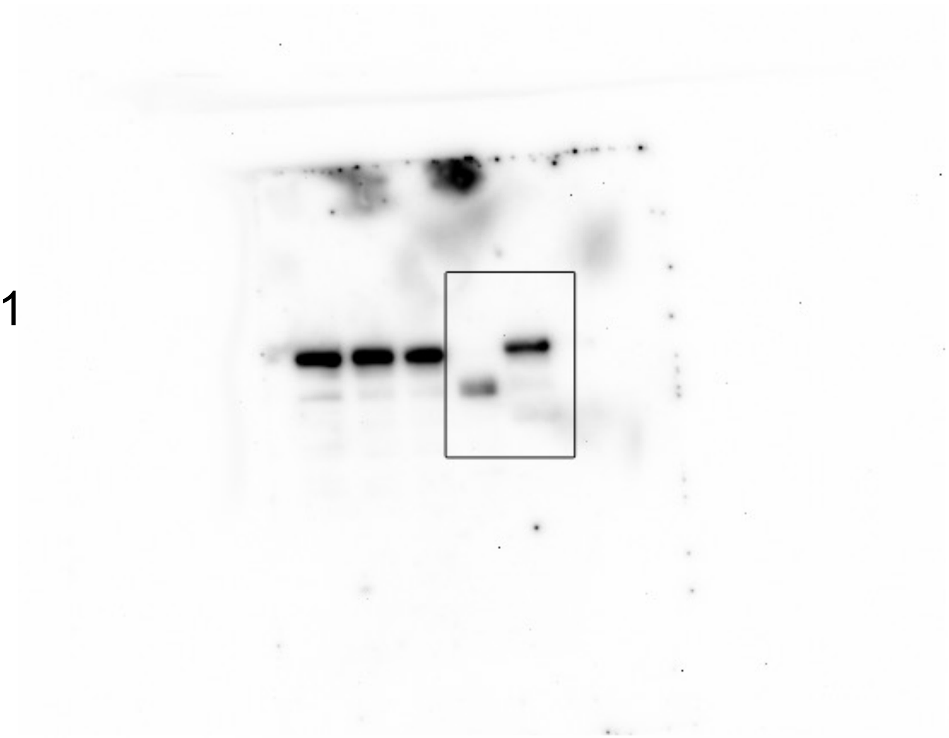

Actin

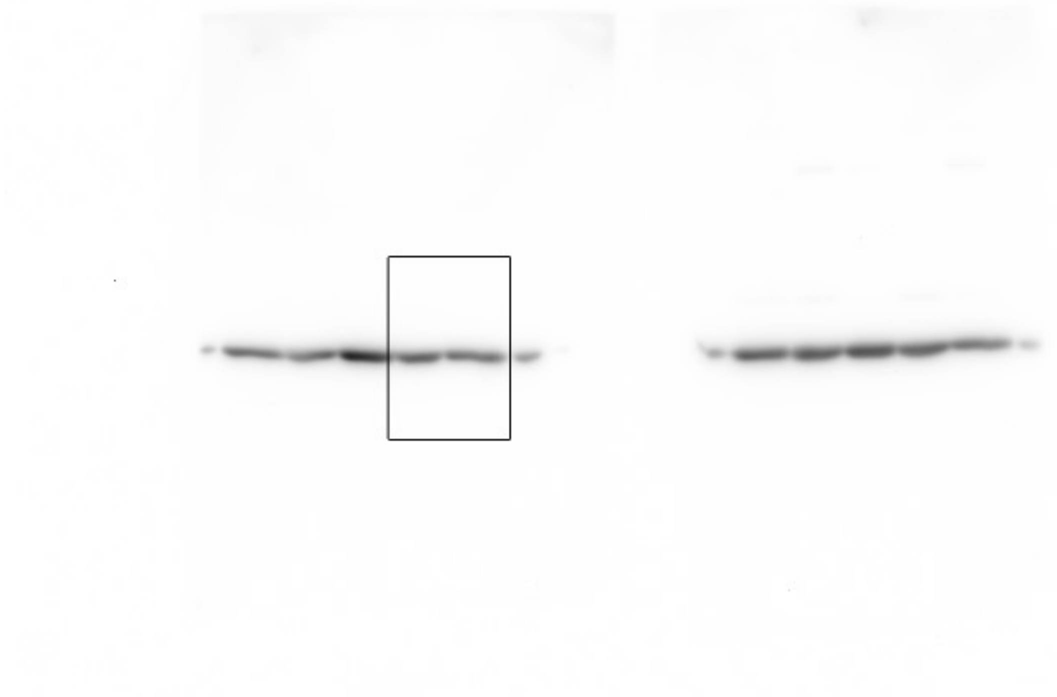

Supplement: Figure 4—figure supplement 1—source data 1. [file elife-80854-fig4-figsupp1-data1.pdf]
