# Supplementary material for: MLL3 regulates the CDKN2A tumor suppressor locus in liver cancer
Source: eLife. 2023 Jun 1;12:e80854. doi: 10.7554/eLife.80854 (PMC10279454; doi:10.7554/eLife.80854)

Figure 5—figure supplement 2B Source files

Myc

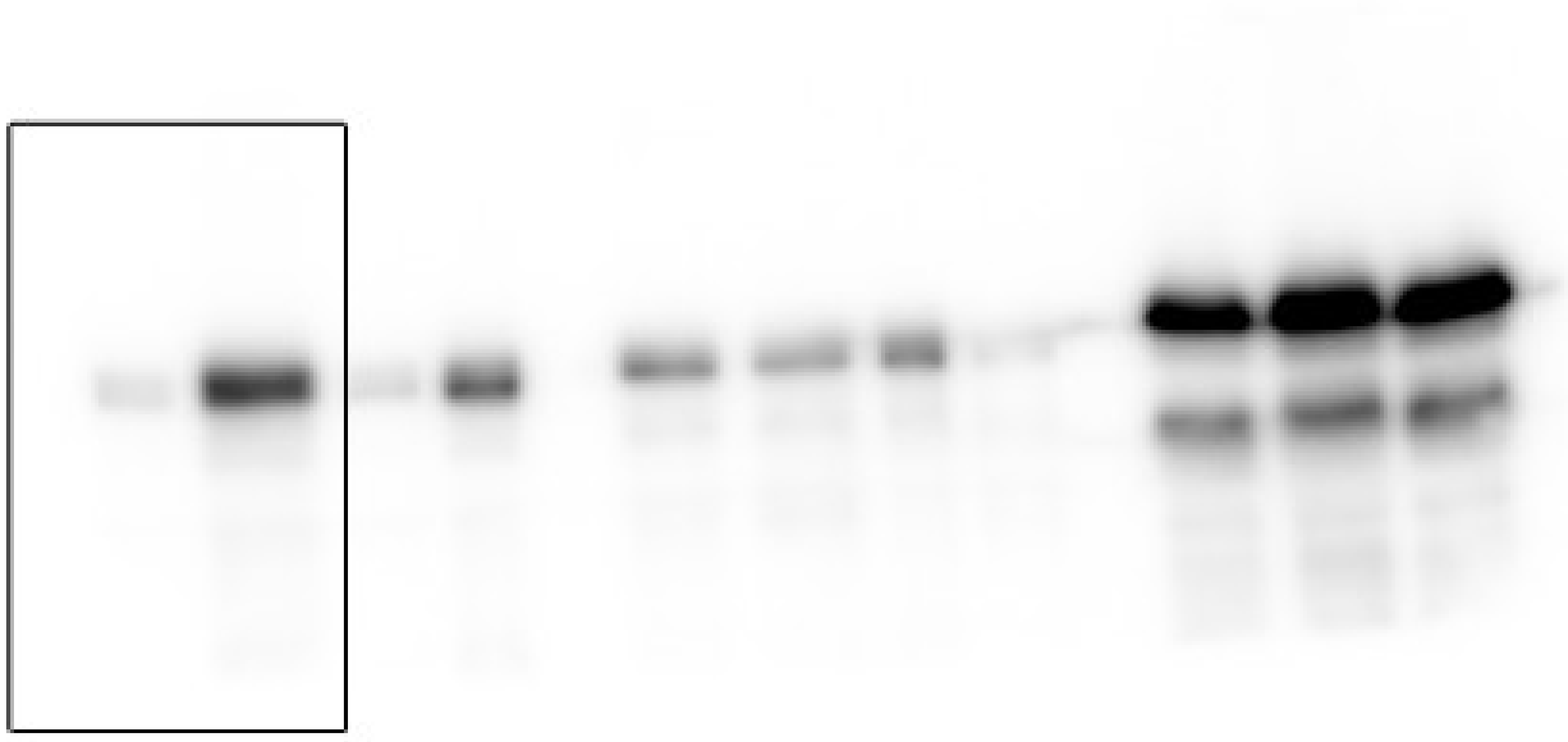

$p19^{Arf}$

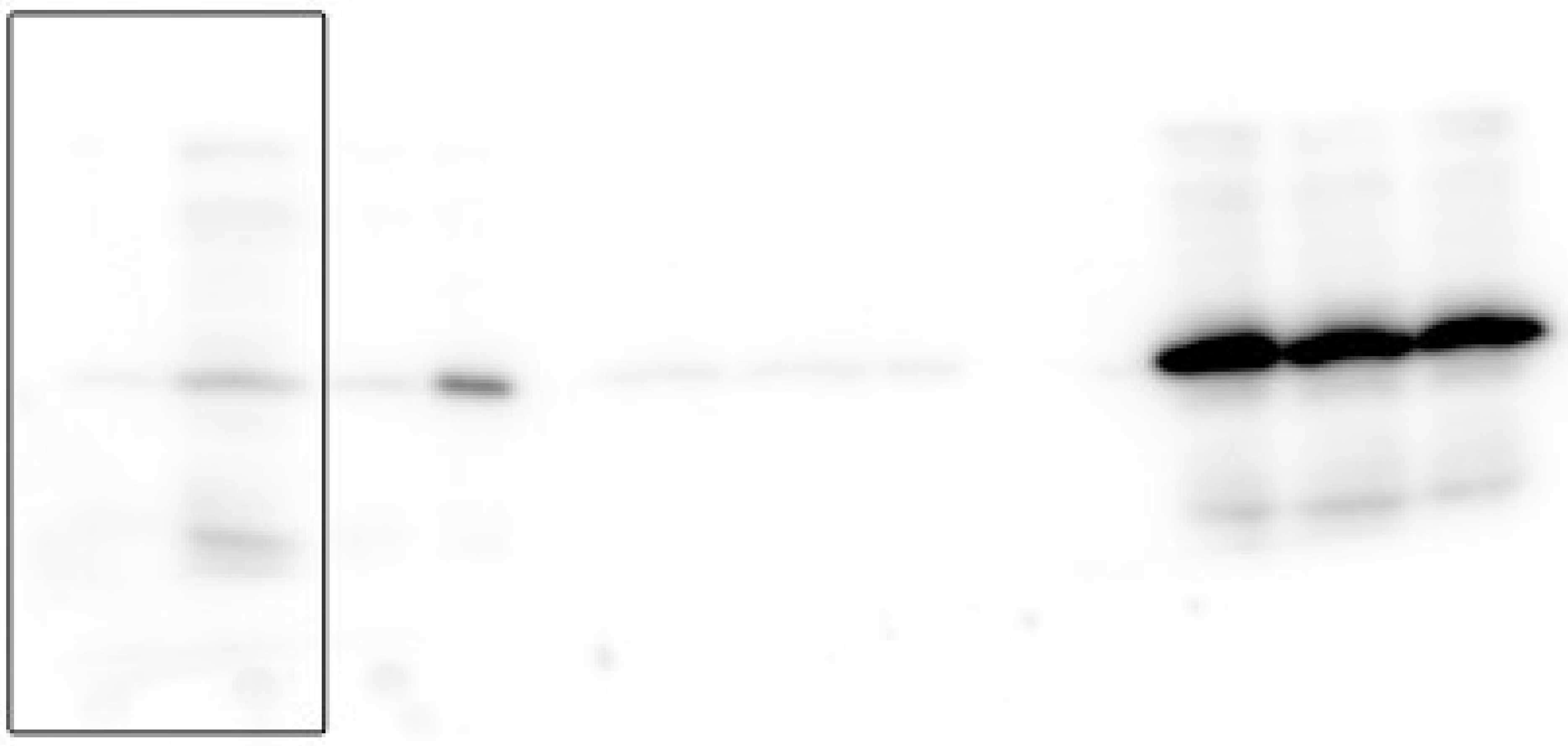

Figure 5—figure supplement 2B Source files

p16^{Ink4a}

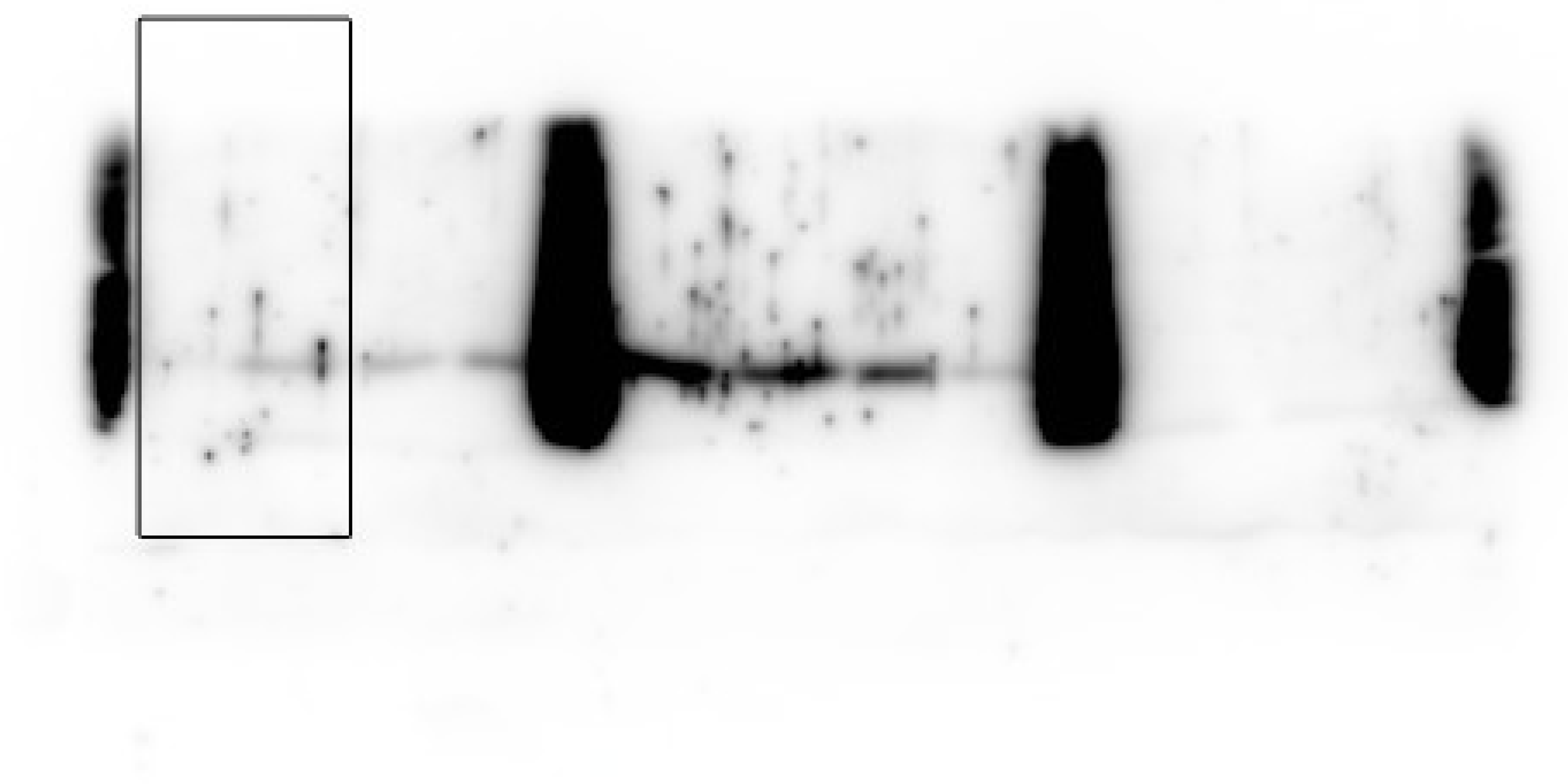

Actin

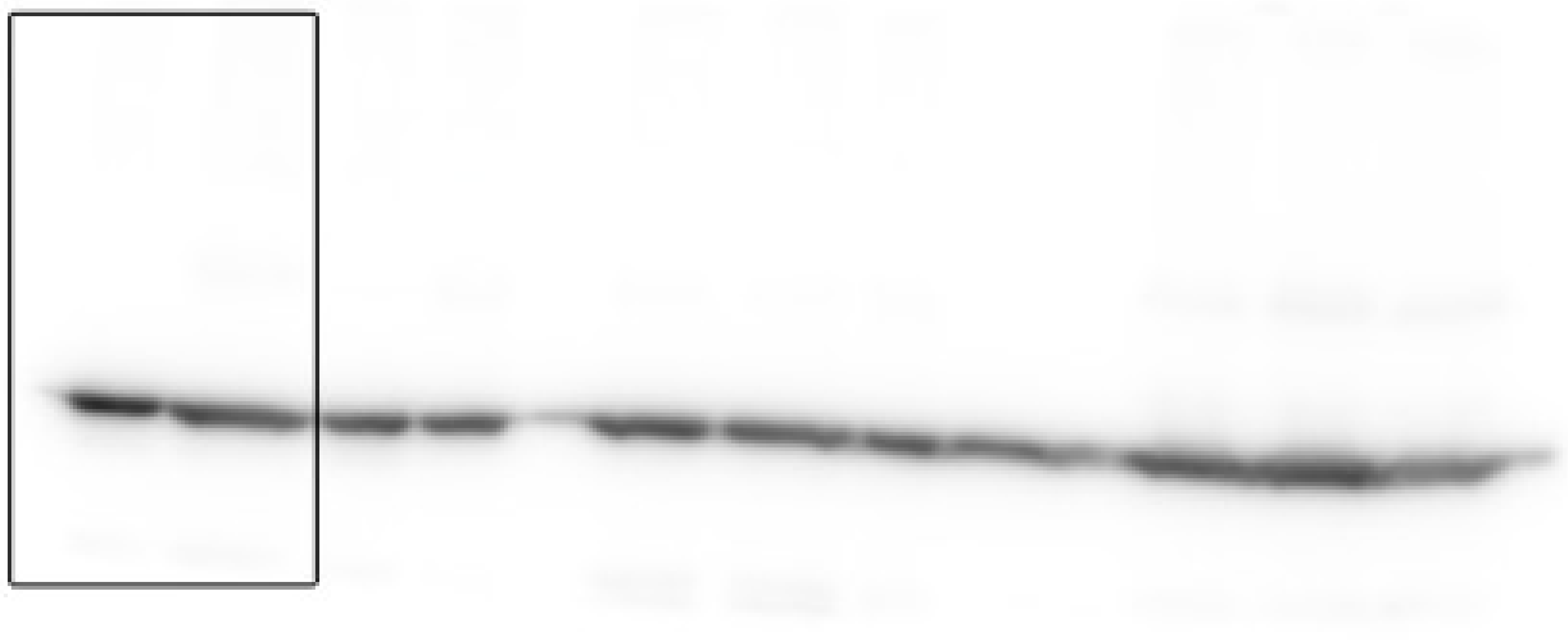

Figure 5—figure supplement 2F Source files – left panel

p19^{Arf}

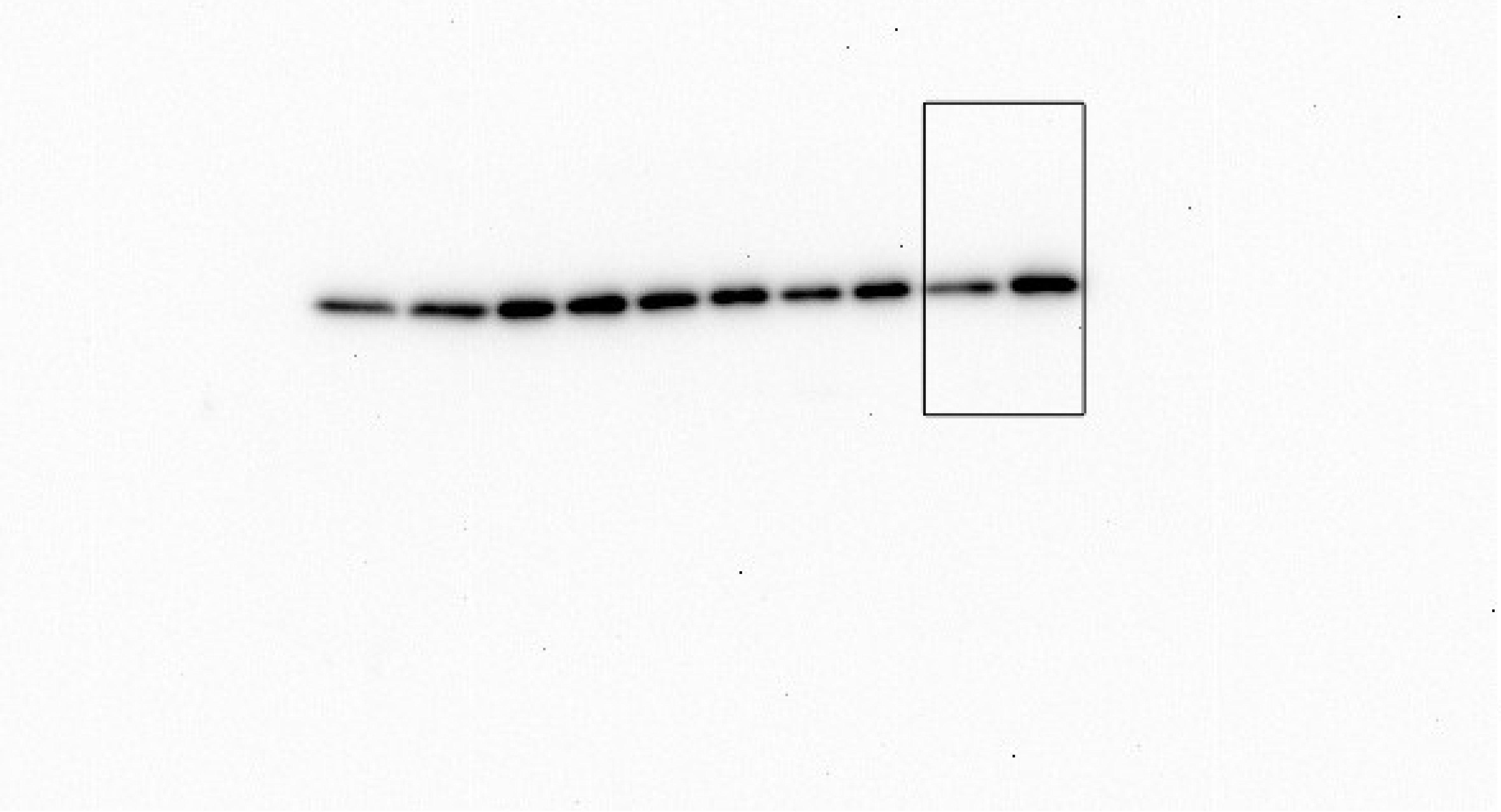

p16^{Ink4a}

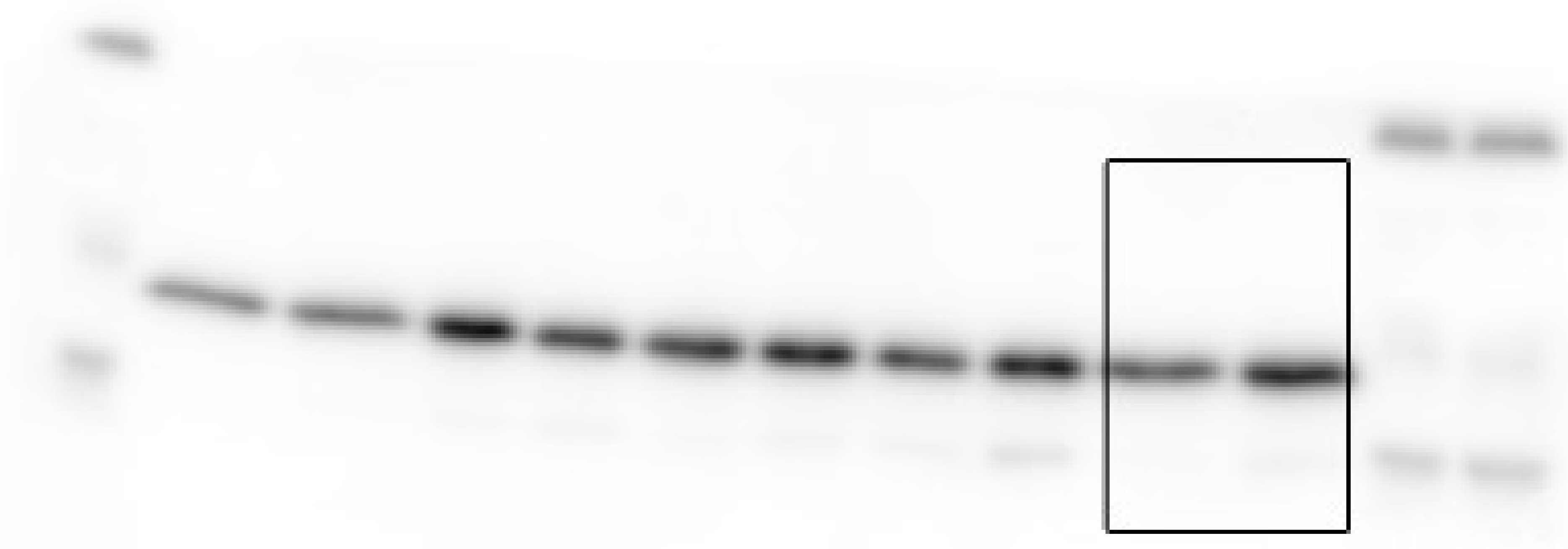

Actin

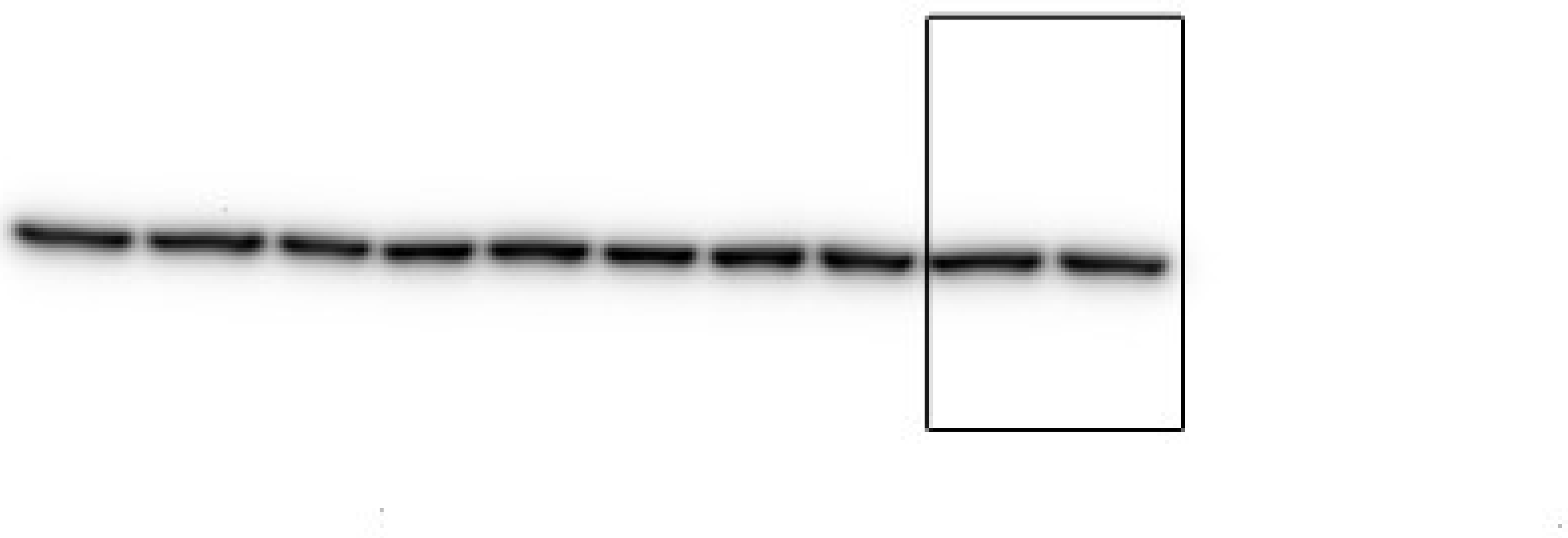

Figure 5—figure supplement 2F Source files – right panel

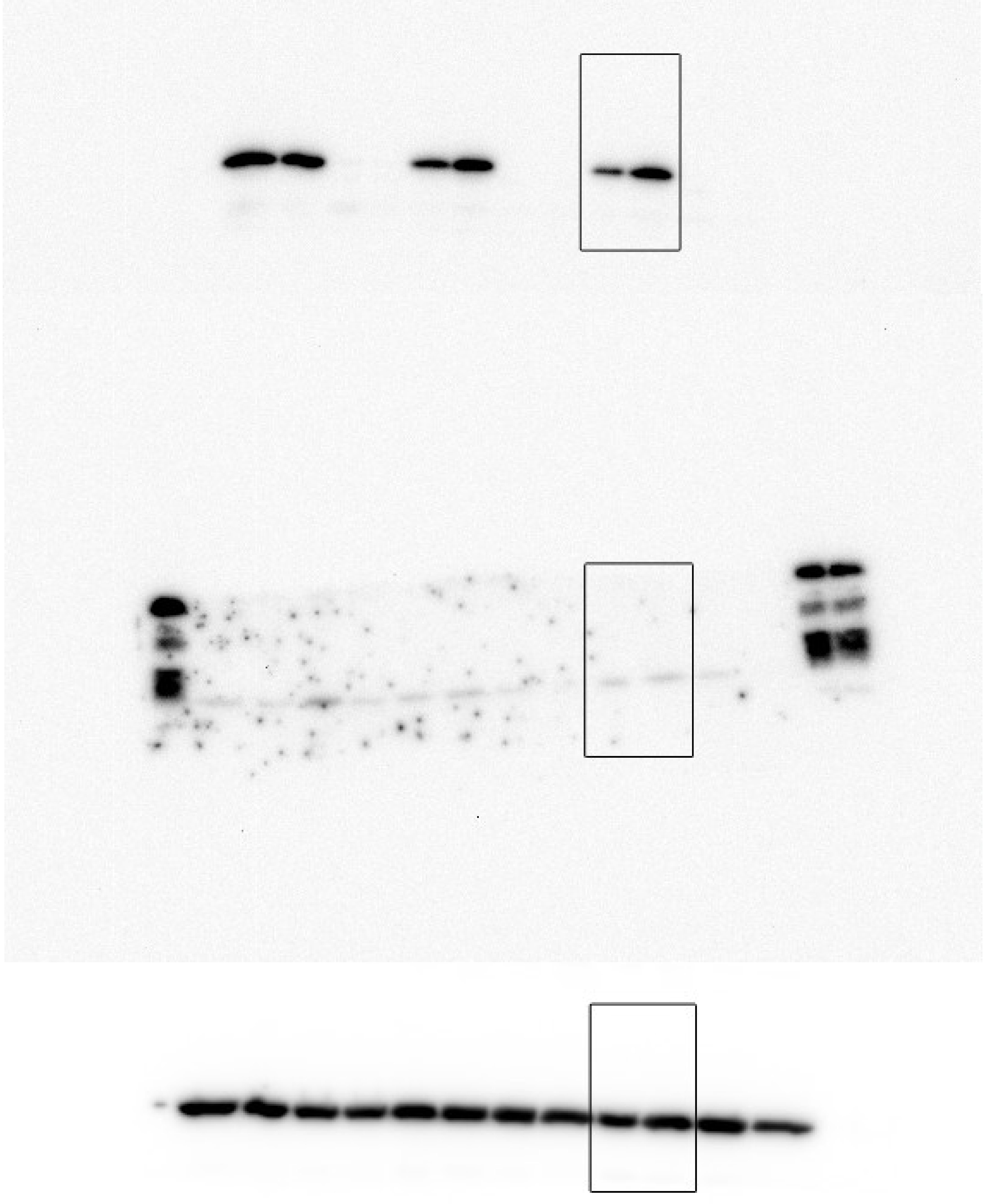

Figure 5—figure supplement 2G Source files

$p19^{Arf}$

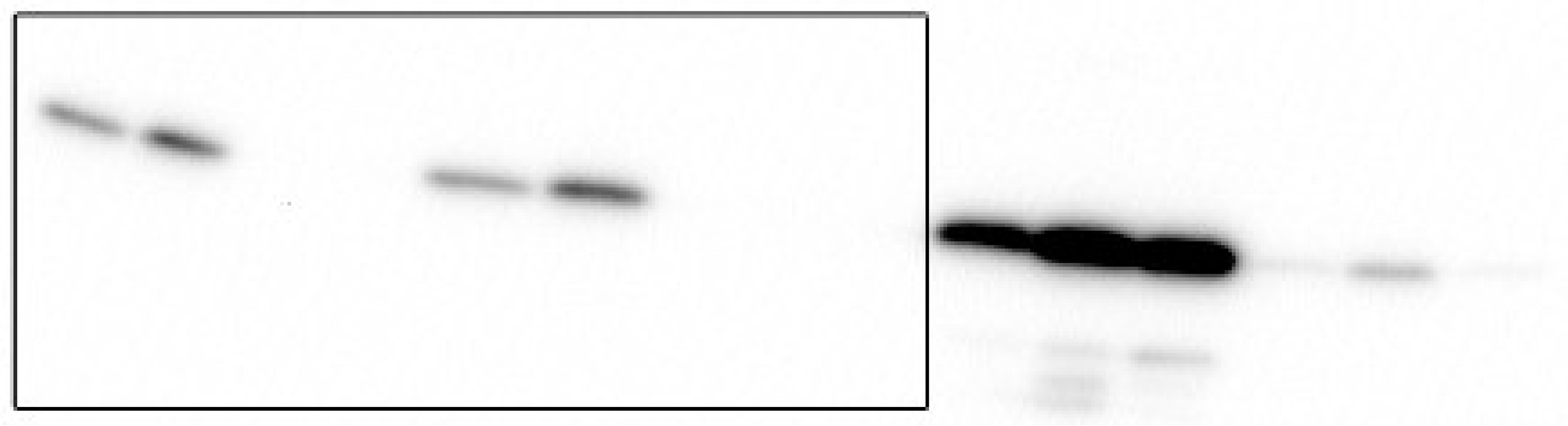

$p16^{Ink4a}$

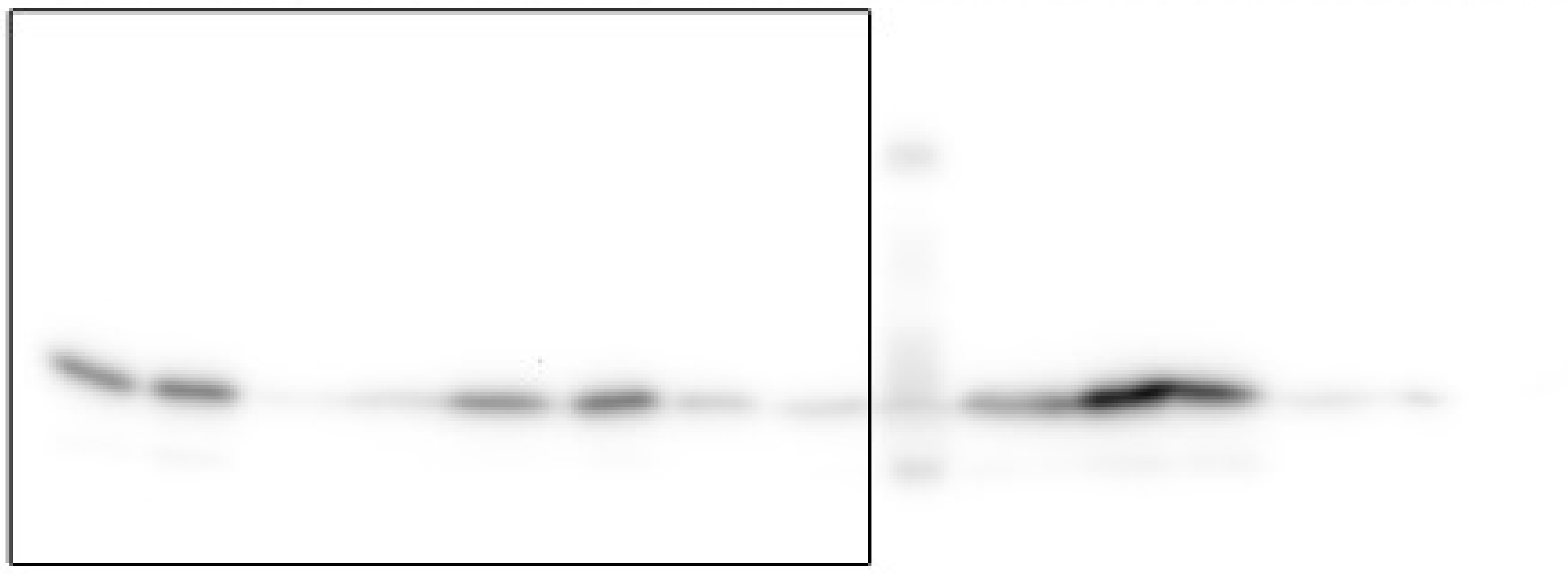

Actin

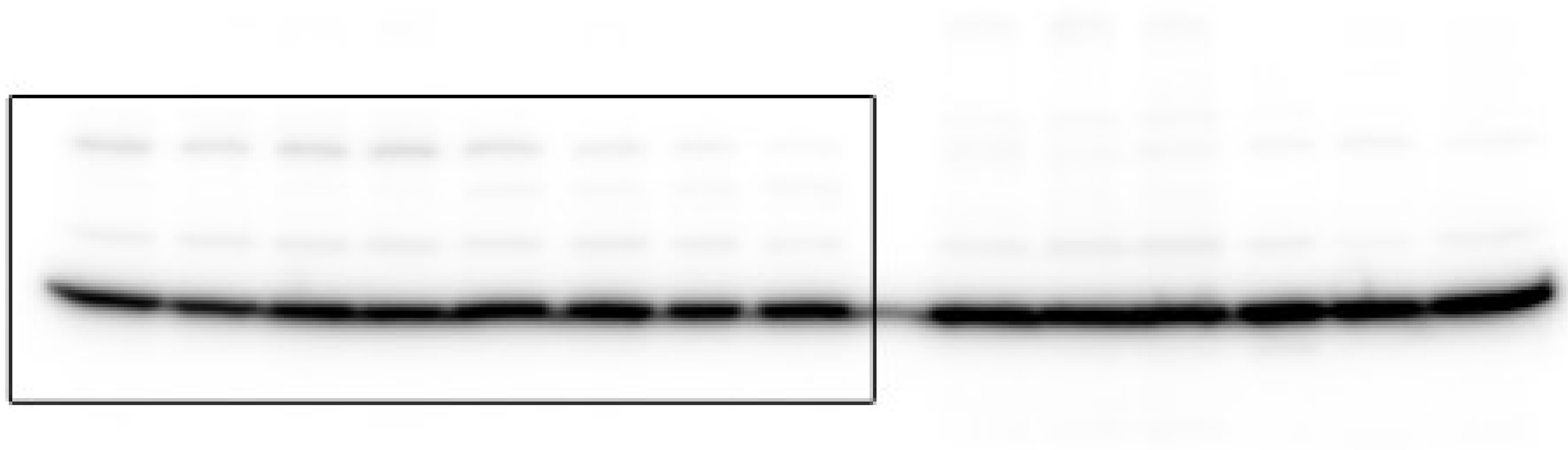

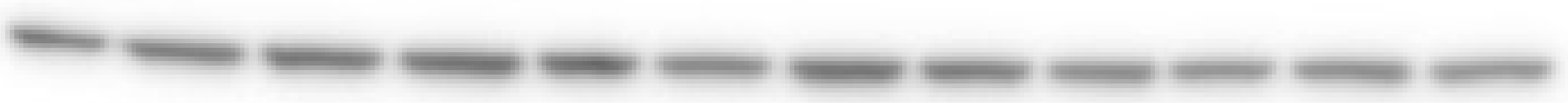

Supplement: Figure 5—figure supplement 2—source data 1. [file elife-80854-fig5-figsupp2-data1.pdf]
