# Supplementary material for: MLL3 regulates the CDKN2A tumor suppressor locus in liver cancer
Source: eLife. 2023 Jun 1;12:e80854. doi: 10.7554/eLife.80854 (PMC10279454; doi:10.7554/eLife.80854)
Supplement: Supplementary file 1. [file elife-80854-supp1.pdf]

Supplementary File 1

sgRNA sequences

sg*Trp53* (mouse)	ACCCTGTCACCGAGACCCC
sg*Kmt2c*.1 (mouse)	AATCAGTGCCAACCAATGGC
sg*Kmt2c*.2 (mouse)	CAGGTTGAAGACAAAAAGTA
sg*Chrom8* (mouse)	ACATTTCTTTCCCCACTGG
sg*GFP*	GAATAGCTCAGAGGCCGAGG
sg*KMT2C*.1 (Human, for CRISPRA)	CCCGCGCGTCAGGCCCGTC
sg*KMT2C*.2 (Human, for CRISPRA)	CAGGCCCGTCAGGCCCGGG

shRNA sequences

sh*Renilla*	AGGAATTATAATGCTTATCT
sh*Kmt2c*.1 (mouse)	GGAGACAAATATGTAGAGTT
sh*Kmt2c*.2 (mouse)	ACCAGTGATCACTTTACTAA
sh*Cdkn2a* (mouse)	AACACAAAGAGCACCCAGCGG

qPCR primers sequences

Actb (mouse)-Forward	GGCTGTATTCCCCTCCATCG
Actb (mouse)-Reverse	CCAGTTGGTAACAATGCCATGT
Cdkn2a-Ink4a (mouse)-Forward	CCATCTGGAGCAGCATGGAGT
Cdkn2a-Ink4a (mouse)-Reverse	ACGTGAACGTTGCCCATCATC
Cdkn2a-Arf (mouse)-Forward	CGCTTCTCACCTCGCTTGTC
Cdkn2a-Arf (mouse)-Reverse	CAGTGACCAAGAACCTGCGA
Kmt2c (mouse)-Forward	CAGGAGGGCCTGCAAGATAC
Kmt2c (mouse)-Reverse	TATCCTCCGGTTGGAGCTGA
ACTB (Human)-Forward	AAGAGCTACGAGCTGCCTGA
ACTB (Human)-Reverse	TCCATGCCCAGGAAGGAAGG
CDKN2A-INK4A (Human)-Forward	GAGCAGCATGGAGCCTTCGG
CDKN2A-INK4A (Human)-Reverse	TGGATCGGCCTCCGACCGTAA
CDKN2A-ARF (Human)-Forward	GCAGGTTCTTGGTGACCCTC
CDKN2A-ARF (Human)-Reverse	TAGACGCTGGCTCCTCAGT
KMT2C (Human)-Forward	GAAACGCTGTAGCCTGTCCT
KMT2C (Human)-Reverse	CCCTGAGTCTCCTTTGGCAG
KMT2D (Human)-Forward	GCCCTTTCTTCAAGGTGGACT
KMT2D (Human)-Reverse	CGGGTTCCGGGCTAAAGAAG
TP53 (Human)-Forward	TGACACGCTTCCCTGGATTG
TP53 (Human)-Reverse	TCATCCATTGCTTGGGACGG

ChIP-qPCR primers sequences

Cdkn2a-Ink4a promoter (mouse)-Forward	GATGGAGCCCGGACTACAGAAG
Cdkn2a-Ink4a promoter (mouse)-Reverse	CTGTTTCAACGCCCAGCTCTC
Cdkn2a-Arf promoter (mouse)-Forward	GACCGTGAAGCCGACCCCTTCAGC
Cdkn2a-Arf promoter (mouse)-Reverse	GGGGTCGCTTTCCCCTTCGG
